# ChatGPT‐4o for Weight Management: Comparison of Different Diet Models

**DOI:** 10.1002/fsn3.70639

**Published:** 2025-07-16

**Authors:** Tugce Ozlu Karahan, Emre Batuhan Kenger

**Affiliations:** ^1^ Faculty of Health Sciences, Department of Nutrition and Dietetics Istanbul Bilgi University Istanbul Turkey

**Keywords:** artificial intelligence, diet, obesity, technology, weight management

## Abstract

In recent years, artificial intelligence (AI) tools such as ChatGPT have emerged as accessible and scalable platforms for generating dietary advice. While ChatGPT has demonstrated potential in providing general nutritional guidance, its capacity to create diet plans tailored to different weight categories and physical activity levels remains underexplored, particularly in comparison across popular dietary (ketogenic and intermittent fasting) models. This study aimed to evaluate the nutritional adequacy and variability of diet plans generated by ChatGPT‐4o for weight management. ChatGPT‐4o generated diet plans for 18 individuals (9 males, 9 females) representing overweight, class I, and class II obesity at varying physical activity levels. Fifty‐four menus were created across three dietary models and analyzed for energy, macro‐, and micronutrient content using the BeBiS nutritional analysis software. Diet variability was also assessed through repeated prompts over three different periods. The ketogenic diets produced by AI had significantly higher energy and saturated fat contents than other models (*p* < 0.05). Regardless of prompt, AI often produced low‐carbohydrate, high‐fat diets. The menus created by ChatGPT had significantly higher fat, saturated fat, and protein content but lower carbohydrate content compared to the dietitian menus (*p* < 0.05). Micronutrient analysis showed frequent inadequacy in calcium, potassium, and vitamin B1. Notably, menu content showed temporal inconsistencies, particularly in intermittent fasting and ketogenic diets. While ChatGPT‐4o shows promise in generating basic dietary models, concerns remain about its nutritional precision, consistency, and safety. The results revealed the necessity of professional supervision in AI‐assisted nutrition planning.

## Graphical abstract

Our results revealed the effects of diet plans created by AI in weight management. As technology improves, further research into the application of AI in nutritional therapy is warranted. The graphical abstract is created by the authors via BioRender (https://www.biorender.com/).

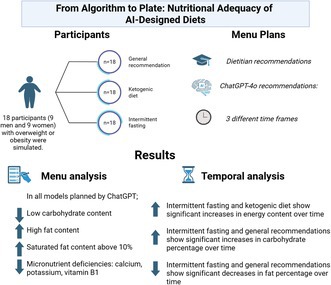

**Abbreviations**
AA: arachidonic acid
AI: artificial intelligence
BeBiS: nutrition information system (Ebispro, Turkish version)
BMI: body mass index
LDL: low‐density lipoprotein
PAL: physical activity level
RDA: recommended dietary allowance
T2D: type 2 diabetes
WHO: World Health Organization

## 1 Introduction

Obesity is a chronic disease characterized by an excessive increase in adipose tissue in the body (Sarma et al. 2021). Obesity, which is increasing daily, is now considered a pandemic (Ahmed and Konje 2023). Obesity, which used to be seen as a problem only in developed countries, has now become a problem in undeveloped/developing countries (Dritsaki and Dritsaki 2023). Obesity, which is thought to affect one billion people by 2030, increases the risk of mortality and morbidity. Increased body mass index (BMI) has been associated with an increased prevalence of chronic diseases such as type 2 diabetes (T2D), hypertension, metabolic syndrome, and chronic renal failure (Sun et al. 2022). It was reported that the risk of all‐cause mortality increased by 21%–108% in participants with BMI ≥ 30 kg/m^2^ (Visaria and Setoguchi 2023).

Lifestyle changes, pharmacological treatment, and surgical options are used in the treatment of overweight and obesity. The most important lifestyle modification is nutrition. The main target in nutrition is diets that provide weight loss by reducing energy intake (Cornier 2022). However, various diet models, such as the ketogenic diet and intermittent fasting, are applied for weight loss (Becker et al. 2021). The ketogenic diet is a dietary model characterized by low carbohydrate (5%–10% or < 50 g/day) and high‐fat consumption. Although the ketogenic diet emerged as a therapeutic method in children with resistant epilepsy (Kossoff et al. 2018), it has recently been used for weight loss. The ketogenic diet has been found to be an effective method to achieve weight loss in individuals with overweight/obesity over a period of 4 weeks to 12 months (Choi et al. 2020). It is stated that the ketogenic diet reduces insulin levels, stimulates fatty acid metabolism, increases energy expenditure with a thermal effect, increases satiety hormone levels, and reduces oxidative stress and inflammation in the mechanism of action on weight loss (Zhu et al. 2022).

Intermittent fasting is a diet model in which the timing of energy intake is at the forefront with energy restriction. This diet model has different applications. There are many different models of intermittent fasting, including time‐restricted feeding and alternate‐day fasting. The 16/8 diet is a common example of time‐restricted feeding with a 16‐h fasting window. In contrast, the 5:2 diet is an alternate‐day fasting protocol in which individuals severely restrict their energy intake (400–600 kcal) two days per week (Kang et al. 2022). There are various reasons for the rapid increase in the popularity of intermittent fasting. The ease of implementation and the fact that individuals do not drastically change their current eating patterns are among these reasons (Varady et al. 2021). In an umbrella‐review study including 11 meta‐analyses examining the effect of intermittent fasting in individuals with overweight/obesity, it was found that it had positive effects on anthropometric and cardiometabolic results (Patikorn et al. 2021). Intermittent fasting is thought to be effective in weight loss by regulating circadian metabolism, increasing satiety, and reducing oxidative stress (Zang et al. 2022).

Artificial intelligence (AI) applications that enable personalized programs in healthcare services are increasing rapidly today (Kim et al. 2024). ChatGPT, an AI‐supported chatbot used in this context, is designed to respond to various questions and topics, from simple queries to complex discussions (Islam et al. 2023). Recently, studies on the role of ChatGPT in weight management have been published. In a study examining the dietary recommendations given by ChatGPT in chronic diseases, it was reported that 70% of the responses were acceptable (Ponzo et al. 2024). In another study, using demographic information such as weight, height, and age, the dietary recommendations given by ChatGPT in individuals with obesity, diabetes, and cardiovascular disease were examined. As a result, it was found that ChatGPT models have the potential to provide personalized nutrition recommendations (Papastratis et al. 2024). However, it is also stated that there are various disadvantages in using ChatGPT, such as not understanding the context of the patient's unique situation, lack of emotional intelligence, privacy and security concerns, and lack of any responsible person (Arslan 2023). While recent studies have explored the ability of AI tools like ChatGPT to generate diet plans with broad nutritional adequacy, most have focused on general weight loss advice or individual clinical cases without comparing AI outputs across multiple obesity levels or dietary models. Additionally, existing research has mainly overlooked whether AI‐generated diets can maintain consistency over time or adapt based on physical activity levels. This study aims to address these critical gaps by analyzing ChatGPT‐4o's ability to generate diet plans for individuals with different BMI classifications and activity levels across three common diet models. It further evaluates these plans' nutritional completeness, variability, and temporal consistency. By doing so, this research provides original insights into the practical reliability and limitations of AI in personalized weight management and nutritional counseling.

## 2 Materials and Methods

### 2.1 Study Design and Objectives

This study aimed to assess the ability of ChatGPT‐4o to generate nutritionally appropriate diet plans for individuals with varying degrees of obesity and physical activity levels based on three dietary models: general recommendations, ketogenic diet, and intermittent fasting.

### 2.2 Participant Simulation

The mean height values for males and females were 176 and 161 cm, respectively. For weights, BMI > 25 kg/m^2^ (overweight), BMI > 30 kg/m^2^ (class I obesity), and BMI > 35 kg/m^2^ (class II obesity) were targeted by using values of 71, 84, 97 kg for females and 85, 101, 116 kg for males, respectively. Age was taken as a constant value of 20 for each individual to prevent the possibility of an increase in chronic diseases that can be seen with age to be predicted by AI. The World Health Organization (WHO) recommendations were used in the classification of BMI (WHO 2010). For each condition, physical activity level (PAL) was determined in three categories: sedentary, low‐level active, and active, and a total of 54 prompts in three different dietary contents (general recommendations, ketogenic diet, intermittent fasting) were entered for 18 individuals (Table 1).

**TABLE 1:** Profile characteristics from individuals with overweight/obesity.

No	Sex	Age	Height (m)	Weight (kg)	Body mass index (kg/m^2^)	Physical activity level
1	Female	20	1.61	71	27.39	Sedantary
2	Female	20	1.61	71	27.39	Low active
3	Female	20	1.61	71	27.39	Active
4	Female	20	1.61	84	32.41	Sedantary
5	Female	20	1.61	84	32.41	Low active
6	Female	20	1.61	84	32.41	Active
7	Female	20	1.61	97	37.42	Sedantary
8	Female	20	1.61	97	37.42	Low active
9	Female	20	1.61	97	37.42	Active
10	Male	20	1.76	85	27.44	Sedantary
11	Male	20	1.76	85	27.44	Low active
12	Male	20	1.76	85	27.44	Active
13	Male	20	1.76	101	32.61	Sedantary
14	Male	20	1.76	101	32.61	Low active
15	Male	20	1.76	101	32.61	Active
16	Male	20	1.76	116	37.45	Sedantary
17	Male	20	1.76	116	37.45	Low active
18	Male	20	1.76	116	37.45	Active

### 2.3 Prompting Approach

The three prompts (general recommendations, ketogenic diet, intermittent fasting) created with ChatGPT‐4o were conducted in English using a new session for each individual, making all conversations independent. The language and sentence structure of the prompts were simple enough for individuals to consult a health professional, as opposed to well‐crafted prompts that may be useful for professional advice. The prompts were repeated in three different periods to determine the consistency of the diet plans prepared by ChatGPT‐4o. Due to the frequent updating of AI tools, it has been stated that a two‐week period will provide consistency in the evaluation by anticipating short‐term fluctuations in responses and the risk of change (Bayram and Ozturkcan 2024). The first prompt entry was completed in August 2024, the second was completed approximately 3 months later in November, and the third was completed 2 weeks later in December. Based on this information, long‐term (3 months) and short‐term (2 weeks) intervals were left to determine the possible variability between the prompts. The prompt levels entered into ChatGPT‐4o for each individual created by the researchers are shown in Table 2.

**TABLE 2:** Prompts samples used for each individual.

	General recommendation	Ketogenic	Intermittent fasting
Sample 1	Can you plan a menu for a 20‐year‐old 161 cm tall, 71 kg female individual with a sedentary activity level and weight loss goal?	Can you plan a ketogenic menu for a 20‐year‐old 161 cm tall, 71 kg female individual with sedentary activity level and weight loss goal?	Can you plan a menu suitable for intermittent fasting for a 20‐year‐old 161 cm tall, 71 kg female individual with sedentary activity level and weight loss goal?
Sample 2	Can you plan a menu for a 20‐year‐old 161 cm tall, 71 kg female individual with a low activity level and weight loss goal?	Can you plan a ketogenic menu for a 20‐year‐old 161 cm tall, 71 kg female individual with a low activity level and weight loss goal?	Can you plan a menu suitable for intermittent fasting for a 20‐year‐old 161 cm tall, 71 kg female individual with a low activity level and weight loss goal?
Sample 3	Can you plan a menu for a 20‐year‐old 161 cm tall, 71 kg female individual with an active level and weight loss goal?	Can you plan a ketogenic menu for a 20‐year‐old 161 cm tall, 71 kg female individual with an active level and weight loss goal?	Can you plan a menu suitable for intermittent fasting for a 20‐year‐old 161 cm tall, 71 kg female individual with an active level and weight loss goal?

### 2.4 Analysis of the Same Prompts by Professional Dietitians

For each prompt condition entered into ChatGPT, professional dietitians (T.O.K.; E.B.K.) have calculated energy and nutrient content and created menu plans. Energy requirements were determined using the Mifflin St. Jeor equation with an activity factor based on physical activity level. Each participant was placed on a hypocaloric diet of 500 kcal below their estimated energy requirement (McLeod et al. 2023). While planning menus based on general recommendations and intermittent fasting, the macronutrient ratios were set at 50%–55% for carbohydrates, 30%–35% for fats, and 15%–20% for proteins. At this point, the only difference between the two diet types was the timing of nutrient intake. Dietitians planned the menu for the intermittent fasting diet programme so that participants would start their first meal at 12:00 and not eat anything after 20:00, applying the 16/8 method. For the ketogenic diet, energy requirements were calculated in the same way, and the upper limit for daily carbohydrate intake was set at 50 g. Additionally, dietitians ensured that the saturated fat content did not exceed 10% of total energy intake.

### 2.5 Evaluation of Dietary Plans

The menu plans written by ChatGPT and dietitians for all three dietary models were entered into the Nutrition Information System (Ebispro for Windows, Germany; Turkish version/BeBiS 8.1) computer package program, and the average daily energy, macro, and micronutrient contents of the menu plans were calculated. BeBIS is a program developed to analyze the energy and nutrients consumed by individuals and the content of foods consumed in Turkish society. The BeBIS program contains more than 130 nutrient data, including energy, macro, and micronutrients specific to the grams of foods consumed (cooked or uncooked), including beverages, packaged foods, and Turkish cultural dishes (with recipes and measurements). Using this database performs energy and nutrient analyses specific to the amounts consumed by individuals. In addition, micronutrient contents for all menu plans were analyzed regarding the daily requirements of male and female individuals of the same age and compared with Recommended Dietary Allowance (RDA) values (National Research Council 1989).

### 2.6 Statistical Analysis

The data obtained were statistically evaluated using the SPSS 28.0 package program. Statistical significance was accepted as *p* < 0.05 in all analyses. The data conformity to normal distribution was checked by Kolmogorov‐Smirnov test. Mean and standard deviation values were included in descriptive statistics. A one‐way ANOVA test was applied to analyze the menus planned by ChatGPT and dietitians according to diet types. Differences between categories were determined by the post hoc Tukey test. In addition, the differences between ChatGPT and dietitians were evaluated using an independent samples *t*‐test. The temporal change of the planned dietary patterns was determined by repeated measures analysis of variance.

## 3 Results

### 3.1 Content Analysis of Menus Planned by ChatGPT

A total of 54 diet plans, including general recommendation, ketogenic diet, and intermittent fasting, were analyzed by ChatGPT‐4o for 18 individuals (9 women and 9 men). When the sample menus are evaluated in terms of content (Table 3), it is observed that they predominantly feature neutral and universally acceptable choices in terms of taste compatibility and cultural acceptability. The sample menus generated by ChatGPT include breakfast, lunch, and dinner, as well as intermediate snacks. The meals commonly feature basic and widely consumed food items such as grilled chicken or salmon, green salad, yoghurt, boiled vegetables, eggs, nuts, and whole‐grain bread. Most of the dishes appeared practical and home‐preparable, with limited use of processed or hard‐to‐access specialty ingredients. While meal timing was not specified in the general recommendation and ketogenic diet menus, clear time allocations were provided in the intermittent fasting model. In these cases, the meals adhered to the 16:8 time‐restricted feeding pattern, with designated eating windows at 12:00, 15:00, 17:00, and 20:00.

**TABLE 3:** Sample representation of menus created by ChatGPT.

General recommendation	Ketogenic	Intermittent fasting
Breakfast 1 slice of whole wheat bread 2 boiled eggs 1 medium tomato and cucumber 1 cup of unsweetened green tea Snack 1 small apple Lunch 100 g grilled chicken breast 1 serving of olive oil green salad 1/2 cup of boiled brown rice Snack 10 almonds Dinner 150 g of grilled salmon 1 cup of boiled broccoli Prepared with 1 teaspoon of olive oil Snack 1 cup of low‐fat yoghurt	Breakfast Omelette made with 2 eggs 1/2 avocado 1 slice of bacon 1 cup of unsweetened coffee Snack 10 almonds Lunch 150 g grilled chicken breast 2 cups of olive oil green salad Snack 1 slice of full‐fat white cheese Dinner 200 g grilled salmon 1 cup of boiled broccoli prepared with 1 teaspoon of olive oil Snack 1 cup of full‐fat yoghurt	12:00—Lunch 150 g grilled chicken breast 2 cups olive oil green salad 1/2 avocado 15:00—Snack 1 cup full‐fat yoghurt 10 almonds 17:00—Dinner 200 g grilled salmon 1 cup of broccoli cooked in 1 teaspoon of olive oil 20:00—Snack 1 slice of whole wheat bread 1 slice of white cheese 1 small apple

### 3.2 Energy and Nutrient Analyses of Menus Planned by Dietitians and ChatGPT

The ketogenic diet menus generated by ChatGPT had the highest mean energy content (2180.1 ± 573.7 kcal), followed by intermittent fasting (1811.3 ± 317.8 kcal) and general recommendations (1797.4 ± 477.2 kcal) (*p* = 0.027). In contrast, the menus prepared by dietitians showed no significant differences in energy content across diet types, maintaining a consistent caloric level for all three models (*p* = 0.995).

Analysis of the macronutrient distribution in ChatGPT‐generated menus revealed that the ketogenic diet group had significantly higher protein (g), fat (g), and fat percentage (%), and lower carbohydrate content (g and %) as expected (*p* < 0.001). Saturated fat intake was highest in the ketogenic group (15.6% ± 0.8%) but also exceeded the recommended limit of 10% in the intermittent fasting (12.4% ± 1.8%) and general recommendation (10.9% ± 1.1%) menus (*p* < 0.001). In contrast, saturated fat content in dietitian‐prepared menus was consistently kept below 10% across all diet models (*p* = 0.131).

Regarding fiber, no significant difference was observed among the diet types in ChatGPT menus (*p* = 0.863). However, the fiber content of the dietitian‐prepared menus, particularly for the general and intermittent fasting diets, was significantly higher than that generated by ChatGPT (*p* < 0.001).

In dietitian‐generated general and intermittent fasting menus, the percentage of energy from protein was maintained at approximately 16%. Conversely, the same menus created by ChatGPT had significantly higher protein percentages, averaging 27%–28% (*p* < 0.001). Similarly, carbohydrate percentages in dietitian menus were around 52%, while ChatGPT's menus provided substantially lower values—approximately 23% and 16% for general and intermittent fasting diets, respectively (*p* < 0.001).

In terms of micronutrients, the general and intermittent fasting menus generated by ChatGPT were found to contain significantly lower levels of calcium, magnesium, vitamin B1, iron, zinc, vitamin A, and vitamin C compared to those developed by dietitians (*p* < 0.05) (Table 4).

**TABLE 4:** Distribution of energy and nutrients in menus planned by ChatGPT and dietitians according to diet types.

	General recommendation	Ketogenic	Intermittent fasting	P^1^
Energy (kcal)				
ChatGPT	1797.4 ± 477.2	2180.1 ± 573.7	1811.3 ± 317.8	**0.027** ^a^
Dietitian recommendation	2084.2 ± 434.1	2071.5 ± 427.3	2084.0 ± 431.7	0.995
*p* ^2^	0.068	0.524	**0.038**	
Protein (g)				
ChatGPT	122.2 ± 22.0	140.7 ± 13.9	119.0 ± 11.5	**< 0.001** [Link], ^b^
Dietitian recommendation	82.6 ± 17.6	145.8 ± 72.7	82.8 ± 17.7	**< 0.001** [Link], ^b^
*p* ^2^	**< 0.001**	0.775	**< 0.001**	
Protein (%)				
ChatGPT	28.4 ± 2.9	27.3 ± 4.7	27.3 ± 2.0	0.565
Dietitian recommendation	16.3 ± 0.51	27.3 ± 8.7	16.4 ± 0.50	**< 0.001**
*p* ^2^	**< 0.001**	0.981	**< 0.001**	
Fat (g)				
ChatGPT	97.7 ± 28.7	163.4 ± 54.2	115.8 ± 28.6	**< 0.001** [Link], ^b^
Dietitian recommendation	72.3 ± 15.5	140.8 ± 15.7	72.6 ± 15.2	**< 0.001** [Link], ^b^
*p* ^2^	**0.002**	0.098	**< 0.001**	
Fat (%)				
ChatGPT	48.2 ± 4.2	65.4 ± 4.4	56.5 ± 4.1	**< 0.001** [Link], ^a^, ^b^
Dietitian recommendation	31.2 ± 1.2	62.0 ± 7.0	31.5 ± 1.3	**< 0.001** [Link], ^b^
*p* ^2^	**< 0.001**	0.088	**< 0.001**	
Carbohydrate (g)				
ChatGPT	104.2 ± 39.7	37.5 ± 11.5	72.5 ± 17.9	**< 0.001** [Link], ^a^, ^b^
Dietitian recommendation	264.3 ± 54.2	49.7 ± 1.2	263.7 ± 54.7	**< 0.001** [Link], ^b^
*p* ^2^	**< 0.001**	**< 0.001**	**< 0.001**	
Carbohydrate (%)				
ChatGPT	23.3 ± 3.5	7.1 ± 0.6	16.2 ± 3.6	**< 0.001** [Link], ^a^, ^b^
Dietitian recommendation	52.4 ± 1.2	10.2 ± 2.0	52.3 ± 1.1	**< 0.001** [Link], ^b^
*p* ^2^	**< 0.001**	**< 0.001**	**< 0.001**	
Fiber (g)				
ChatGPT	26.5 ± 11.3	27.2 ± 11.0	28.4 ± 8.4	0.863
Dietitian recommendation	39.1 ± 19.3	22.8 ± 3.6	39.2 ± 19.5	**< 0.001** [Link], ^b^
*p* ^2^	**< 0.001**	0.112	**< 0.001**	
Fructose (g)				
ChatGPT	13.5 ± 3.8	1.4 ± 0.5	9.7 ± 3.0	**< 0.001** [Link], ^a^, ^b^
Dietitian recommendation	31.0 ± 8.4	2.1 ± 0.4	30.8 ± 8.2	**< 0.001** [Link], ^b^
*p* ^2^	**< 0.001**	**< 0.001**	**< 0.001**	
Saturated fat (%)				
ChatGPT	10.9 ± 1.1	15.6 ± 0.8	12.4 ± 1.8	**< 0.001** [Link], ^a^, ^b^
Dietitian recommendation	9.5 ± 0.8	8.9 ± 1.4	9.5 ± 0.7	0.131
*p* ^2^	**< 0.001**	**< 0.001**	**< 0.001**	
Monounsaturated fatty acid (g)				
ChatGPT	43.0 ± 14.9	70.8 ± 21.5	51.1 ± 10.7	**< 0.001** [Link], ^b^
Dietitian recommendation	18.3 ± 3.7	68.3 ± 4.5	18.4 ± 3.7	**< 0.001** [Link], ^b^
*p* ^2^	**< 0.001**	0.631	**< 0.001**	
Polyunsaturated fatty acid (g)				
ChatGPT	27.9 ± 7.3	46.9 ± 22.9	36.1 ± 17.4	**0.006** ^a^
Dietitian recommendation	26.4 ± 3.8	43.4 ± 7.5	26.5 ± 5.9	**< 0.001** [Link], ^b^
*p* ^2^	0.527	0.546	**0.034**	
Omega 3 (g)				
ChatGPT	8.4 ± 3.1	12.0 ± 5.8	10.2 ± 5.1	0.097
Dietitian recommendation	9.6 ± 2.5	10.5 ± 2.2	9.7 ± 2.4	0.526
*p* ^2^	0.224	0.297	0.677	
Omega 6 (g)				
ChatGPT	19.3 ± 4.2	34.7 ± 17.2	25.6 ± 13.4	**0.003** ^a^
Dietitian recommendation	16.6 ± 3.9	32.8 ± 5.4	16.7 ± 3.6	**< 0.001** [Link], ^b^
*p* ^2^	0.062	0.652	**0.011**	
Omega 6/Omega 3				
ChatGPT	2.4 ± 0.5	2.9 ± 0.3	2.7 ± 1.1	0.17
Dietitian recommendation	1.8 ± 0.7	3.2 ± 0.3	1.9 ± 0.6	**< 0.001** [Link], ^b^
*p* ^2^	**0.007**	**0.017**	**0.005**	
Sodium (mg)				
ChatGPT	1843.5 ± 266.5	2087.0 ± 407.6	1613.0 ± 1427.2	0.273
Dietitian recommendation	2059.6 ± 1089.4	533.2 ± 114.1	2020.7 ± 1073.1	**< 0.001** ^**α†**^
*p* ^2^	**< 0.001**	**< 0.001**	**< 0.001**	
Potassium (mg)				
ChatGPT	3394.2 ± 929.4	4329.8 ± 963.0	3435.6 ± 325.4	**0.001** [Link], ^b^
Dietitian recommendation	3999.7 ± 744.1	3416.5 ± 677.6	3994.1 ± 748.4	**0.028** [Link], ^b^
*p* ^2^	**0.038**	**< 0.001**	**0.006**	
Calcium (mg)				
ChatGPT	706.8 ± 162.5	801.9 ± 187.4	654.6 ± 61.0	**0.014** ^c^
Dietitian recommendation	1393.4 ± 219.3	781.8 ± 183.6	1395.0 ± 223.0	**< 0.001** [Link], ^b^
*p* ^2^	**< 0.001**	0.746	**< 0.001**	
Magnesium (mg)				
ChatGPT	396.2 ± 135.1	425.6 ± 153.1	404.9 ± 137.8	0.816
Dietitian recommendation	608.3 ± 135.4	457.6 ± 112.2	608.9 ± 136.6	**< 0.001** [Link], ^b^
*p* ^2^	**< 0.001**	0.481	**< 0.001**	
Iron (mg)				
ChatGPT	11.3 ± 3.1	15.4 ± 1.9	9.1 ± 1.8	**< 0.001** [Link], ^a^, ^b^
Dietitian recommendation	21.3 ± 5.1	12.1 ± 4.9	21.4 ± 5.2	**< 0.001** [Link], ^b^
*p* ^2^	**< 0.001**	**0.013**	**< 0.001**	
Zinc (mg)				
ChatGPT	9.1 ± 2.2	10.3 ± 3.3	8.2 ± 1.9	0.058
Dietitian recommendation	15.8 ± 3.8	14.2 ± 7.2	15.9 ± 3.9	0.529
*p* ^2^	**< 0.001**	**0.047**	**< 0.001**	
Vitamin A (mcg)				
ChatGPT	978.2 ± 346.0	1265.6 ± 525.8	557.3 ± 242.4	**< 0.001** ^a^, ^b^
Dietitian recommendation	1254.3 ± 221.0	481.1 ± 157.5	1247.5 ± 220.1	**< 0.001** [Link], ^b^
*p* ^2^	**0.007**	**< 0.001**	**< 0.001**	
Vitamin E (mg)				
ChatGPT	26.6 ± 4.8	30.5 ± 5.5	25.9 ± 7.2	0.056
Dietitian recommendation	18.8 ± 5.4	45.4 ± 8.6	18.8 ± 5.3	**< 0.001** [Link], ^b^
*p* ^2^	**< 0.001**	**< 0.001**	**0.002**	
Vitamin B1 (mg)				
ChatGPT	1.1 ± 0.2	1.1 ± 0.3	1.03 ± 0.2	0.644
Dietitian recommendation	2.0 ± 0.4	1.2 ± 0.2	2.0 ± 0.4	**< 0.001** [Link], ^b^
*p* ^2^	**< 0.001**	0.994	**< 0.001**	
Vitamin B12 (mcg)				
ChatGPT	10.41 ± 1.10	12.29 ± 0.79	9.35 ± 0.29	**< 0.001** [Link], ^a^, ^b^
Dietitian recommendation	3.9 ± 0.9	7.1 ± 1.3	4.0 ± 0.9	**< 0.001** [Link], ^b^
*p* ^2^	**< 0.001**	**< 0.001**	**< 0.001**	
Vitamin C (mg)				
ChatGPT	140.99 ± 35.39	69.32 ± 22.12	143.15 ± 13.55	**< 0.001** [Link], ^b^
Dietitian recommendation	250.8 ± 53.4	50.9 ± 1.3	249.8 ± 54.7	**< 0.001** [Link], ^b^
*p* ^2^	**< 0.001**	**0.001**	**< 0.001**	

*Note:* P^1^, One Way Anova, significant difference *p* ≤ 0.05; P^2^, Independent sample *t* test, significant difference *p* ≤ 0.05, statistical significance indicated in bold.

^a^ Significant difference between general recommendation and ketogenic diet.

^b^ Significant difference between general recommendation and intermittent fasting.

^c^ Significant difference between ketogenic diet and intermittent fasting.

### 3.3 Percentage of Menus Planned by ChatGPT That Meet RDA Recommendations

**FIGURE 1:**
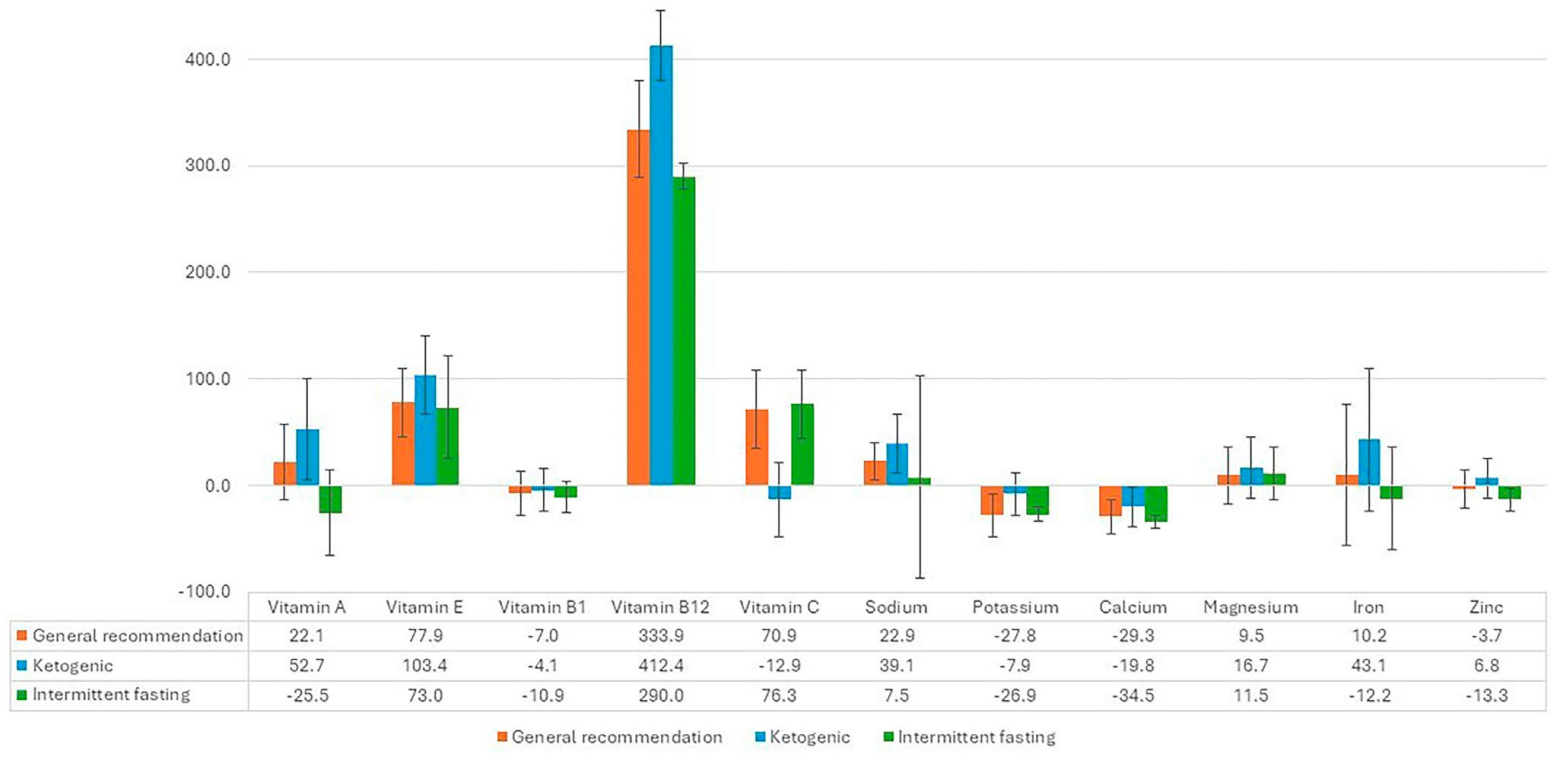
Deviation values (%) of micronutrient contents in menus planned by ChatGPT from RDA recommendations. RDA, recommended dietary allowance.

In addition to these findings, RDA coverage percentages of micronutrient content of three different diet plans were determined. The groups' RDA percentages of vitamin A, B12, C, potassium, calcium, iron, and zinc significantly differed (*p* < 0.05). The RDA percentages of vitamin A, B12, iron, and zinc were lowest in the intermittent fasting plan, the vitamin C RDA percentage was lowest in the ketogenic diet plan, and potassium and calcium RDA percentages were highest in the ketogenic diet plan (*p* < 0.05). No significant differences were found for vitamin E, vitamin B1, sodium, and magnesium (*p* > 0.05) (Figure 1).

### 3.4 Analysis of Menus Planned by ChatGPT According to Different BMI Levels and PAL Levels

**FIGURE 2:**
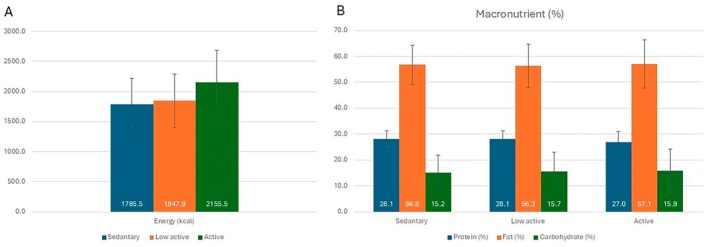
Distribution of energy (A) and macronutrients (B) in menus planned by ChatGPT according to PAL classifications.

Energy and nutrient distributions were also analyzed in terms of different BMI levels and PAL levels. It was observed that the energy, macronutrient, and micronutrient contents of the diet plans were similar according to overweight, class 1, and class 2 obesity, and there was no significant difference between the groups (*p* > 0.05) (Table 5). In terms of PAL levels, it was determined that the energy and macronutrient distributions of the diets were similar in sedentary, low active, and active groups (*p* > 0.05) (Figure 2).

**TABLE 5:** Distribution of energy and nutrients in menus planned by ChatGPT according to BMI classifications.

	Overweight	Obesity class I	Obesity class II	*p* ^a^
Energy (kcal)	1794.1 ± 417.9	2018.0 ± 574.1	1976.7 ± 472.4	0.356
Protein (g)	121.0 ± 16.3	129.8 ± 21.0	131.2 ± 18.1	0.218
Protein (%)	28.1 ± 3.3	27.2 ± 3.4	27.8 ± 3.5	0.704
Fat (g)	115.4 ± 39.2	133.1 ± 55.3	128.4 ± 47.4	0.522
Fat (%)	56.1 ± 7.3	57.2 ± 8.9	56.7 ± 8.7	0.924
Carbohydrate (g)	67.0 ± 32.5	74.4 ± 43.7	72.9 ± 37.2	0.830
Carbohydrate (%)	15.6 ± 6.6	15.6 ± 8.0	15.5 ± 7.5	1.000
Fiber (g)	26.3 ± 10.4	28.2 ± 10.4	27.7 ± 10.0	0.845
Fructose (g)	8.3 ± 5.2	8.2 ± 6.6	8.1 ± 5.7	0.993
Saturated fat (%)	26.6 ± 8.2	28.9 ± 10.5	28.5 ± 9.1	0.870
Monounsaturated fatty acid (g)	50.9 ± 16.9	57.8 ± 22.6	56.2 ± 20.2	0.559
Polyunsaturated fatty acid (g)	32.8 ± 14.2	40.3 ± 22.4	37.6 ± 18.4	0.480
Omega 3 (g)	9.6 ± 4.1	10.8 ± 5.1	10.3 ± 4.9	0.778
Omega 6 (g)	23.1 ± 10.8	29.4 ± 17.0	27.1 ± 13.9	0.410
Omega 6/Omega 3	2.5 ± 0.8	2.8 ± 0.6	2.7 ± 0.7	0.581
Sodium (mg)	1859.9 ± 911.2	1802.8 ± 866.1	1880.9 ± 899.4	0.964
Potassium (mg)	3580.0 ± 880.3	3815.0 ± 971.6	3765.0 ± 858.6	0.716
Calcium (mg)	688.2 ± 125.8	734.0 ± 170.4	741.1 ± 174.5	0.557
Magnesium (mg)	382.2 ± 123.7	429.6 ± 158.1	414.9 ± 139.9	0.592
Iron (mg)	11.0 ± 3.2	12.4 ± 3.7	12.3 ± 3.5	0.453
Zinc (mg)	8.7 ± 2.3	9.6 ± 3.0	9.4 ± 2.6	0.588
Vitamin A (mcg)	870.0 ± 440.8	926.7 ± 495.6	1004.5 ± 523.5	0.710
Vitamin E (mg)	26.7 ± 5.9	28.3 ± 6.5	28.0 ± 6.3	0.727
Vitamin B1 (mg)	1.0 ± 0.2	1.1 ± 0.2	1.1 ± 0.2	0.643
Vitamin B12 (mcg)	10.5 ± 1.4	10.6 ± 1.4	10.8 ± 1.5	0.823
Vitamin C (mg)	116.0 ± 41.4	120.5 ± 46.6	116.8 ± 41.9	0.946

^a^ One Way Anova, significant difference *p* ≤ 0.05.

### 3.5 Temporal Analysis of Menus Planned by ChatGPT

**FIGURE 3:**
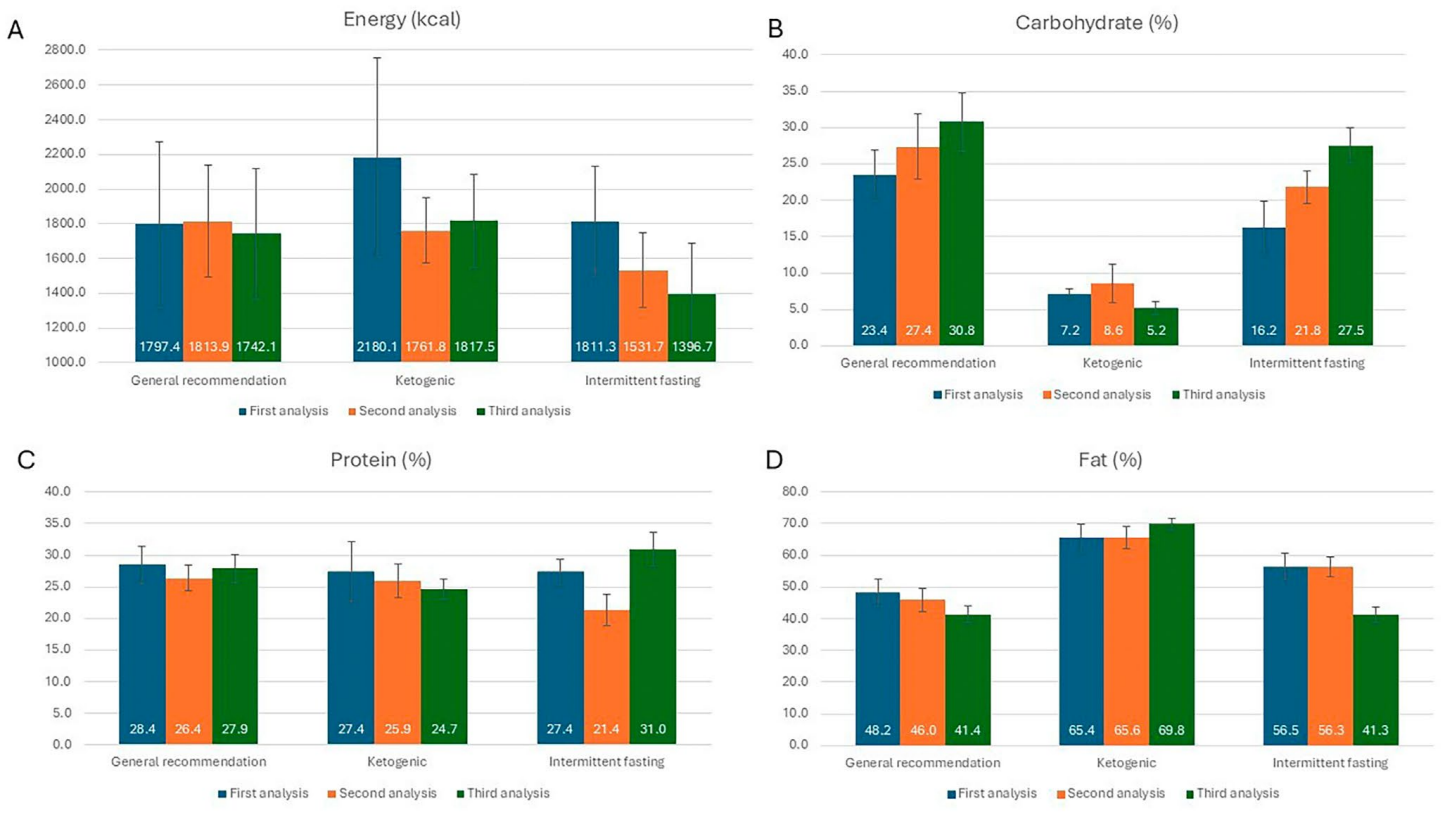
Temporal changes in the energy and macronutrient content of menus planned by ChatGPT. Mean daily energy intake (A) and percentages of energy from carbohydrate (B), protein (C), and fat (D) for the menu plans based on measurements at three different times.

In addition, the same prompts specific to the same individuals were entered into the ChatGPT‐4o tool at three different time points. The percentages of energy and macronutrients from energy were analyzed. Although similarity was observed between the three measurements in terms of energy for general recommendations (*p* = 0.674), the mean energy of 2180.1 ± 573.5 kcal in the first measurement of the ketogenic diet group decreased to 1761.8 ± 189.2 kcal in the second measurement (*p* < 0.001). A continuous decrease was observed after the first measurement (from 1811.3 ± 317.8 kcal to 1396.7 ± 293.2 kcal) for intermittent fasting (*p* < 0.001). When the percentage of macronutrients from energy was analyzed, a continuous increase in the percentage of carbohydrates (27.5% ± 2.4% from 16.2% ± 3.6%; *p* < 0.001) and a decrease in the percentage of fat (41.3% ± 2.3% from 56.5% ± 4.1%; *p* < 0.001) was observed after the first measurement for intermittent fasting. For the general recommendations, a similar situation was observed for carbohydrate (from 23.4% ± 3.5% to 30.8% ± 3.9%; *p* < 0.001) and fat percentage (from 48.2% ± 4.3% to 41.4% ± 2.6%; *p* < 0.001). For the ketogenic diet, there was an increase in fat percentage from the first to the third measurement (from 65.4% ± 4.5% to 69.8% ± 1.7%; *p* < 0.001). Considering the percentage of protein from energy, significant temporal changes were found in the ketogenic diet (*p* = 0.022) and intermittent fasting group (*p* < 0.001), except for the general recommendation (Figure 3).

## 4 Discussion

Today, overweight/obesity, whose prevalence continues to increase, is one of the most common and complex health problems of modern societies and requires multifaceted approaches in its treatment. In recent years, the role of AI technologies in treating overweight/obesity has been investigated. In addition, intermittent fasting and ketogenic diet models have been frequently used in weight management. In our study, different diet models created by AI for weight management were analyzed, and their potential effects were discussed.

In our study, when the diet contents were analyzed, AI prepared the energy content of the ketogenic diet higher than the other diets (general: 1797.4 kcal, ketogenic: 2180.1 kcal, intermittent fasting: 1811.3). It was found that AI tended to prepare a low carbohydrate content even if the ketogenic diet prompt was not given (general: 23.4%, ketogenic: 7.2%, intermittent fasting: 16.2%). In contrast, in another study, it was reported that omnivores created a diet with a fat content of 33% (Hieronimus et al. 2024). It is emphasized that diets with low carbohydrate content can be effective in weight management (Silverii et al. 2022). However, it has also been shown to be effective in the management of diseases such as non‐alcoholic fatty liver (Hu et al. 2023), T2D (Goldenberg et al. 2021), and polycystic ovary syndrome (Mei et al. 2022). However, there are studies indicating that diets with low carbohydrate content increase the risk of low‐density lipoprotein (LDL) (Chawla et al. 2020), cancer (Cai et al. 2022) and mortality (Zhao et al. 2023). The long‐term effects of low‐carbohydrate diets and their impact on clinical endpoints [e.g., myocardial infarction, stroke, and total mortality] are largely unknown (Dong et al. 2020). In this context, users should be aware of the potential long‐term effects of the low‐carbohydrate content of AI‐supported dietary models. On the other hand, when a comparison is made, it is observed that the menus created by dietitians maintained similar energy levels across all diet models and achieved a macronutrient distribution consistent with recommended targets. This finding suggests that AI‐based systems may be limited in their ability to tailor dietary plans to individual energy requirements compared to human expertise.

However, the amount of fiber in AI's diet was sufficient (general: 26.5 g, ketogenic: 27.2 g, intermittent fasting: 28.4 g). Despite its low carbohydrate content, it is thought that the adequacy of its fiber content is effective in using non‐starchy vegetables and oil seeds such as almonds, walnuts, and chia in meal planning. In a similar study, AI was shown to prepare a diet model with high fiber content (Hieronimus et al. 2024). Fiber consumption positively affects weight management and diabetes, cardiovascular diseases, and gastrointestinal system diseases (He et al. 2022). Moreover, in terms of dietary fiber, the menus prepared by dietitians—particularly within the general recommendation and intermittent fasting models—provided significantly higher fiber content compared to those generated by ChatGPT. This difference is due to the dietitians' more intentional and balanced inclusion of fiber‐rich foods such as vegetables, legumes, whole grains, and fruits.

Our study found that AI tended to prepare a content high in saturated fat, which was higher in the ketogenic diet (general: 10.9%, ketogenic: 15.6%, intermittent fasting: 12.4%). A diet high in saturated fat is not recommended for weight loss, as it may increase inflammation (Zhou et al. 2020). In addition, it is emphasized that consuming diets high in saturated fatty acids increases the risk of cardiovascular and all‐cause mortality (Zheng et al. 2023). Although saturated fat content is expected to be higher in the ketogenic diet, the preparation of diets with higher saturated fat content than the levels recommended for health (< 10%) in other dietary models (WHO 2023) may have an adverse effect. In contrast, saturated fat intake in the menus planned by dietitians was consistently kept below the 10% threshold. This finding indicates that AI‐assisted dietary plans may have limitations in controlling fat quality. Although AI systems can achieve certain macronutrient distribution targets, they appear to insufficiently account for qualitative differences between saturated and unsaturated fat sources. However, the AI omega 6/omega 3 ratio was within the recommended levels, and there was no significant difference in dietary patterns (general: 2.4, ketogenic: 2.9, intermittent fasting: 2.7). Individuals with overweight and obesity have higher levels of arachidonic acid (AA)‐derived endocannabinoids. Since the omega 6/omega 3 ratio plays a role in both regulating inflammation and modulating appetite, it is recommended that individuals with overweight and obesity reduce their omega‐6 fatty acid intake and increase their omega‐3 fatty acid intake (Simopoulos 2020). Considering the recommendation that the omega 6/omega 3 ratio should be 5/1 or less for reducing inflammation and general health (Sut et al. 2020), AI and dietitian menus seem to comply with this ratio.

The micronutrient contents of the diet plans prepared by AI were mostly above or in accordance with recommendations, except for vitamin B1, calcium, and potassium, which were below the recommendations. When a comparison was made between diets, the highest content of B12 and iron was found in the ketogenic diet. This is thought to be due to the fact that AI prescribes more animal‐based foods. Calcium and potassium consumption recommendations were below the RDA in all three dietary patterns. Similar to our research, AI has established a diet low in calcium and potassium content for omnivores (Hieronimus et al. 2024). Considering the anti‐obesity effects of calcium (Zhang et al. 2019), this content is unsuitable for commands targeting weight loss. In addition, low calcium consumption negatively affects bone health (Rizzoli et al. 2021) and increases cardiovascular risks (Lee et al. 2024). With respect to calcium, the menus generated by ChatGPT—particularly in the general recommendation and intermittent fasting models—contained significantly lower levels compared to those developed by dietitians. This suggests that AI‐based systems may insufficiently address calcium requirements, a micronutrient essential for bone health.

In our study, some differences were found in the energy and macronutrient percentages of the menu plans written by ChatGPT over time. Different reasons can explain this situation. Artificial intelligence tools are constantly retrained with new data sets, and their algorithms are improved. At the same time, the data sets used by AI tools may also change. Updating or changing information in large data sources such as the Internet directly affects the models' responses. In addition, user feedback also plays a role in changing the responses of AI tools. Little is known about how temporal response changes can affect people's perceptions and use of the model (Wang and Yin 2023). Research is needed on the potential consequences of these changes, especially in an important area such as human health.

With all these factors, weight loss is not only about dietary modeling, calculation of energy, and macro/micronutrients. The process of weight loss and its successful maintenance involves complex interactions between behavioral, physiological, environmental, economic, and cognitive/psychosocial factors. It is well known that weight loss maintenance relies on permanent adjustments between behaviors that reduce energy intake and those that increase energy expenditure. Some psychological determinants, such as self‐efficacy (i.e., monitoring one's weight and eating behavior), autonomous motivation, and a positive body image, seem to influence behavior change (Paixão et al. 2020). On the other hand, barriers to weight management are not the same in different socioeconomic statuses. Financial constraints may worsen the person's lifestyle or affect access to the nutrients in the diet plan. Economic factors should be considered in weight management (Mehrabi et al. 2021). However, communication is crucial to the success of the weight loss process. At this point, dietitians are key interventionists in treating overweight and obesity, providing behavioral change and motivation by providing nutrition education with effective communication techniques (Morgan‐Bathke et al. 2023). Although the potential success of AI regarding calculations and dietary models is not ignored, the interaction of individuals with nutritionists is also important.

While this study primarily focused on the quantitative analysis of nutrient content and energy distribution, qualitative factors such as palatability, cultural acceptance, and practical feasibility also play a crucial role in real‐world dietary adherence. When examining the sample menus generated by ChatGPT‐4o, they did not exhibit an overrepresentation of specific national cuisines such as Mexican or Chinese; instead, they predominantly featured broadly acceptable food items such as grilled chicken, steamed vegetables, and yoghurt. The dishes presented were generally international and neutral. This indicates that ChatGPT leans toward common and balanced meals rather than cultural diversity. On the other hand, while the majority of the suggested meals included ingredients that are accessible and feasible for home preparation, certain items such as avocado or full‐fat white cheese may not be readily available to all individuals due to economic or regional limitations. Sociocultural factors and individual habits are important factors that directly affect food selection and dietary plan adoption (Losavio et al. 2023). In this context, the extent to which AI‐generated menus consider factors such as the availability of certain ingredients, meal preparation time, and individual lifestyle restrictions is stated as an important issue (Amiri et al. 2024). These factors should also be taken into account in future studies.

Despite the important findings of this study, it has some limitations. Firstly, although samples from different BMI groups were included, a single age parameter was used, and a single height value was entered for men and women. Second, although the ketogenic diet and intermittent fasting models, which are most used in weight loss, were examined, other diet models were not analyzed. In addition, the number of simulated participants was limited; including a larger and more diverse sample could have increased the generalizability and robustness of the results. Potential bias in large language models toward Western diet patterns is another limitation.

While this study focused on the nutritional composition and consistency of AI‐generated diet plans, future research should take a more applied approach. First, intervention studies are needed to evaluate the real‐world effectiveness of menus created by ChatGPT—specifically, whether individuals following these plans experience meaningful improvements in weight, adherence, and metabolic health outcomes. In addition, it would be valuable to assess ChatGPT's role as a communication and behavior change tool, especially in the context of weight loss. Comparing the motivational and educational impact of AI guidance with that of professional dietitians could provide important insight into the broader applicability of AI in long‐term lifestyle interventions. Furthermore, there is a need for further studies on this subject by increasing the number of samples and adding other diet models.

## 5 Conclusion

This study is the first to examine the content of different nutritional models created by the ever‐developing AI for weight management. While the AI produced structured menus with generally adequate macronutrients and fiber, it frequently exceeded recommended levels of saturated fat—especially in ketogenic plans—and consistently fell short on key micronutrients such as calcium, potassium, and vitamin B1. Despite individualized input on BMI and activity levels, the diet outputs showed minimal variation, and significant inconsistencies were observed over time in repeated prompts, particularly for energy and macronutrient composition. These findings suggest that while ChatGPT‐4o holds potential as a supportive tool in dietary planning, its outputs require professional oversight. AI should complement—not replace—individualized nutrition care led by qualified dietitians. Further refinement and validation of AI models are essential for safe integration into clinical nutrition practice.

## Author Contributions

**Tugce Ozlu Karahan:** conceptualization (equal), data curation (equal), formal analysis (equal), investigation (equal), methodology (equal), writing – original draft (equal), writing – review and editing (equal). **Emre Batuhan Kenger:** conceptualization (equal), data curation (equal), formal analysis (equal), investigation (equal), methodology (equal), writing – original draft (equal), writing – review and editing (equal).

## Conflicts of Interest

The authors declare no conflicts of interest.

## Data Availability

The data of the current research can be accessed via https://osf.io/f9sab/.

## References

[fsn370639-bib-0001] Ahmed, B. , and J. C. Konje . 2023. “The Epidemiology of Obesity in Reproduction.” Best Practice & Research. Clinical Obstetrics & Gynaecology 89: 102342. 10.1016/j.bpobgyn.2023.102342. 37276817

[fsn370639-bib-0002] Amiri, M. , F. Sarani Rad , and J. Li . 2024. “Delighting Palates With AI: Reinforcement Learning's Triumph in Crafting Personalized Meal Plans With High User Acceptance.” Nutrients 16, no. 3: 346. 10.3390/nu16030346. 38337630 PMC10857145

[fsn370639-bib-0003] Arslan, S. 2023. “Exploring the Potential of Chat GPT in Personalized Obesity Treatment.” Annals of Biomedical Engineering 51, no. 9: 1887–1888. 10.1007/s10439-023-03227-9. 37145177

[fsn370639-bib-0004] Bayram, H. M. , and A. Ozturkcan . 2024. “AI Showdown: Info Accuracy on Protein Quality Content in Foods From ChatGPT 3.5, ChatGPT 4, Bard AI and Bing Chat.” British Food Journal 126, no. 9: 3335–3346. 10.1108/BFJ-02-2024-0158.

[fsn370639-bib-0005] Becker, A. , D. Gaballa , M. Roslin , E. Gianos , and J. Kane . 2021. “Novel Nutritional and Dietary Approaches to Weight Loss for the Prevention of Cardiovascular Disease: Ketogenic Diet, Intermittent Fasting, and Bariatric Surgery.” Current Cardiology Reports 23, no. 7: 85. 10.1007/s11886-021-01515-1. 34081228

[fsn370639-bib-0006] Cai, H. , T. Sobue , T. Kitamura , et al. 2022. “Low‐Carbohydrate Diet and Risk of Cancer Incidence: The Japan Public Health Center‐Based Prospective Study.” Cancer Science 113, no. 2: 744–755. 10.1111/cas.15215. 34821435 PMC8819285

[fsn370639-bib-0007] Chawla, S. , F. Tessarolo Silva , S. Amaral Medeiros , R. A. Mekary , and D. Radenkovic . 2020. “The Effect of Low‐Fat and Low‐Carbohydrate Diets on Weight Loss and Lipid Levels: A Systematic Review and Meta‐Analysis.” Nutrients 12, no. 12: 3774. 10.3390/nu12123774. 33317019 PMC7763365

[fsn370639-bib-0008] Choi, Y. J. , S. M. Jeon , and S. Shin . 2020. “Impact of a Ketogenic Diet on Metabolic Parameters in Patients With Obesity or Overweight and With or Without Type 2 Diabetes: A Meta‐Analysis of Randomized Controlled Trials.” Nutrients 12, no. 7: 2005. 10.3390/nu12072005. 32640608 PMC7400909

[fsn370639-bib-0009] Cornier, M. A. 2022. “A Review of Current Guidelines for the Treatment of Obesity.” American Journal of Managed Care 28, no. 15 Suppl: S288–S296. 10.37765/ajmc.2022.89292. 36525676

[fsn370639-bib-0010] Dong, T. , M. Guo , P. Zhang , G. Sun , and B. Chen . 2020. “The Effects of Low‐Carbohydrate Diets on Cardiovascular Risk Factors: A Meta‐Analysis.” PLoS One 15, no. 1: e0225348. 10.1371/journal.pone.0225348. 31935216 PMC6959586

[fsn370639-bib-0011] Dritsaki, M. , and C. Dritsaki . 2023. “The Impact of GDP, Human Development, Unemployment, and Globalization on Obesity.” Asian Economic and Financial Review 13, no. 7: 431–462. 10.55493/5002.v13i7.4799.

[fsn370639-bib-0012] Goldenberg, J. Z. , A. Day , G. D. Brinkworth , et al. 2021. “Efficacy and Safety of Low and Very Low Carbohydrate Diets for Type 2 Diabetes Remission: Systematic Review and Meta‐Analysis of Published and Unpublished Randomized Trial Data.” BMJ (Clinical Research Ed.) 372: m4743. 10.1136/bmj.m4743. PMC7804828 33441384

[fsn370639-bib-0013] He, Y. , B. Wang , L. Wen , et al. 2022. “Effects of Dietary Fiber on Human Health.” Food Science and Human Wellness 11: 1–10. 10.1016/j.fshw.2021.07.001.

[fsn370639-bib-0014] Hieronimus, B. , S. Hammann , and M. C. Podszun . 2024. “Can the AI Tools ChatGPT and Bard Generate Energy, Macro‐ and Micro‐Nutrient Sufficient Meal Plans for Different Dietary Patterns?” Nutrition Research (New York, N.Y.) 128: 105–114. 10.1016/j.nutres.2024.07.002. 39102765

[fsn370639-bib-0015] Hu, C. , R. Huang , R. Li , et al. 2023. “Low‐Carbohydrate and Low‐Fat Diet With Metabolic‐Dysfunction‐Associated Fatty Liver Disease.” Nutrients 15, no. 22: 4763. 10.3390/nu15224763. 38004162 PMC10674227

[fsn370639-bib-0016] Islam, M. R. , T. J. Urmi , R. A. Mosharrafa , M. S. Rahman , and M. F. Kadir . 2023. “Role of ChatGPT in Health Science and Research: A Correspondence Addressing Potential Application.” Health Science Reports 6, no. 10: e1625. 10.1002/hsr2.1625. 37841943 PMC10568002

[fsn370639-bib-0017] Kang, J. , N. A. Ratamess , A. D. Faigenbaum , et al. 2022. “Effect of Time‐Restricted Feeding on Anthropometric, Metabolic, and Fitness Parameters: A Systematic Review.” Journal of the American Nutrition Association 41, no. 8: 810–825. 10.1080/07315724.2021.1958719. 34491139

[fsn370639-bib-0018] Kim, D. W. , J. S. Park , K. Sharma , et al. 2024. “Qualitative Evaluation of Artificial Intelligence‐Generated Weight Management Diet Plans.” Frontiers in Nutrition 11: 1374834. 10.3389/fnut.2024.1374834. 38577160 PMC10991711

[fsn370639-bib-0019] Kossoff, E. H. , B. A. Zupec‐Kania , S. Auvin , et al. 2018. “Optimal Clinical Management of Children Receiving Dietary Therapies for Epilepsy: Updated Recommendations of the International Ketogenic Diet Study Group.” Epilepsia Open 3, no. 2: 175–192. 10.1002/epi4.12225. 29881797 PMC5983110

[fsn370639-bib-0020] Lee, J. K. , T. M. C. Tran , E. Choi , et al. 2024. “Association Between Daily Dietary Calcium Intake and the Risk of Cardiovascular Disease (CVD) in Postmenopausal Korean Women.” Nutrients 16, no. 7: 1043. 10.3390/nu16071043. 38613076 PMC11013752

[fsn370639-bib-0021] Losavio, J. , M. J. Keenan , E. A. Gollub , and H. J. Silver . 2023. “Factors That Predict Weight Loss Success Differ by Diet Intervention Type.” Frontiers in Nutrition 10: 1192747. 10.3389/fnut.2023.1192747. 37599685 PMC10434209

[fsn370639-bib-0022] McLeod, A. , P. Wolf , R. S. Chapkin , et al. 2023. “Design of the Building Research in CRC Prevention (BRIDGE‐CRC) Trial: A 6‐Month, Parallel Group Mediterranean Diet and Weight Loss Randomized Controlled Lifestyle Intervention Targeting the Bile Acid‐Gut Microbiome Axis to Reduce Colorectal Cancer Risk Among African American/Black Adults With Obesity.” Trials 24, no. 1: 113. 10.1186/s13063-023-07115-4. 36793105 PMC9930092

[fsn370639-bib-0023] Mehrabi, F. , N. Ahmaripour , S. Jalali‐Farahani , and P. Amiri . 2021. “Barriers to Weight Management in Pregnant Mothers With Obesity: A Qualitative Study on Mothers With Low Socioeconomic Background.” BMC Pregnancy and Childbirth 21, no. 1: 779. 10.1186/s12884-021-04243-0. 34789171 PMC8597093

[fsn370639-bib-0024] Mei, S. , J. Ding , K. Wang , Z. Ni , and J. Yu . 2022. “Mediterranean Diet Combined With a Low‐Carbohydrate Dietary Pattern in the Treatment of Overweight Polycystic Ovary Syndrome Patients.” Frontiers in Nutrition 9: 876620. 10.3389/fnut.2022.876620. 35445067 PMC9014200

[fsn370639-bib-0025] Morgan‐Bathke, M. , S. D. Baxter , T. M. Halliday , et al. 2023. “Weight Management Interventions Provided by a Dietitian for Adults With Overweight or Obesity: An Evidence Analysis Center Systematic Review and Meta‐Analysis.” Journal of the Academy of Nutrition and Dietetics 123, no. 11: 1621–1661. 10.1016/j.jand.2022.03.014. 35788061 PMC12646720

[fsn370639-bib-0026] National Research Council Subcommittee on the Tenth Edition of the Recommended Dietary A . 1989. “The National Academies Collection: Reports Funded by National Institutes of Health.” In Recommended Dietary Allowances: 10th Edition. National Academies Press (US) Copyright 1989 by the National Academy of Sciences.

[fsn370639-bib-0027] Paixão, C. , C. M. Dias , R. Jorge , et al. 2020. “Successful Weight Loss Maintenance: A Systematic Review of Weight Control Registries.” Obesity Reviews: An Official Journal of the International Association for the Study of Obesity 21, no. 5: e13003. 10.1111/obr.13003. 32048787 PMC9105823

[fsn370639-bib-0028] Papastratis, I. , A. Stergioulas , D. Konstantinidis , P. Daras , and K. Dimitropoulos . 2024. “Can ChatGPT Provide Appropriate Meal Plans for NCD Patients?” Nutrition 121: 112291. 10.1016/j.nut.2023.112291. 38359704

[fsn370639-bib-0029] Patikorn, C. , K. Roubal , S. K. Veettil , et al. 2021. “Intermittent Fasting and Obesity‐Related Health Outcomes: An Umbrella Review of Meta‐Analyses of Randomized Clinical Trials.” JAMA Network Open 4, no. 12: e2139558. 10.1001/jamanetworkopen.2021.39558. 34919135 PMC8683964

[fsn370639-bib-0030] Ponzo, V. , I. Goitre , E. Favaro , et al. 2024. “Is ChatGPT an Effective Tool for Providing Dietary Advice?” Nutrients 16, no. 4: 469. 10.3390/nu16040469. 38398794 PMC10892804

[fsn370639-bib-0031] Rizzoli, R. , E. Biver , and T. C. Brennan‐Speranza . 2021. “Nutritional Intake and Bone Health.” Lancet. Diabetes & Endocrinology 9, no. 9: 606–621. 10.1016/S2213-8587(21)00119-4. 34242583

[fsn370639-bib-0032] Sarma, S. , S. Sockalingam , and S. Dash . 2021. “Obesity as a Multisystem Disease: Trends in Obesity Rates and Obesity‐Related Complications.” Diabetes, Obesity & Metabolism 23, no. Suppl 1: 3–16. 10.1111/dom.14290. 33621415

[fsn370639-bib-0033] Silverii, G. A. , C. Cosentino , F. Santagiuliana , et al. 2022. “Effectiveness of Low‐Carbohydrate Diets for Long‐Term Weight Loss in Obese Individuals: A Meta‐Analysis of Randomized Controlled Trials.” Diabetes, Obesity & Metabolism 24, no. 8: 1458–1468. 10.1111/dom.14709. PMC9546386 35373905

[fsn370639-bib-0034] Simopoulos, A. P. 2020. “Omega‐6 and Omega‐3 Fatty Acids: Endocannabinoids, Genetics and Obesity.” Ocl 27: 7. 10.1051/ocl/2019046.

[fsn370639-bib-0035] Sun, X. , A. F. Yan , Z. Shi , et al. 2022. “Health Consequences of Obesity and Projected Future Obesity Health Burden in China.” Obesity (Silver Spring, Md.) 30, no. 9: 1724–1751. 10.1002/oby.23472. 36000246

[fsn370639-bib-0036] Sut, A. , C. Krzysztof , R. Marcin , and G. Jacek . 2020. “Dietary intake of omega fatty acids and polyphenols and its relationship with the levels of inflammatory markers in men with chronic coronary syndrome after percutaneous coronary intervention.” Kardiologia Polska 78, no. 2: 117–123. 10.33963/KP.15078. 31790083

[fsn370639-bib-0037] Varady, K. A. , S. Cienfuegos , M. Ezpeleta , and K. Gabel . 2021. “Cardiometabolic Benefits of Intermittent Fasting.” Annual Review of Nutrition 41: 333–361. 10.1146/annurev-nutr-052020-041327. 34633860

[fsn370639-bib-0038] Visaria, A. , and S. Setoguchi . 2023. “Body Mass Index and All‐Cause Mortality in a 21st Century U.S. Population: A National Health Interview Survey Analysis.” PLoS One 18, no. 7: e0287218. 10.1371/journal.pone.0287218. 37405977 PMC10321632

[fsn370639-bib-0039] Wang, X. , and M. Yin . 2023. “Watch out for Updates: Understanding the Effects of Model Explanation Updates in Ai‐Assisted Decision Making.” In Proceedings of the 2023 CHI Conference on Human Factors in Computing Systems no. 758: 1–19. 10.1145/3544548.3581366.

[fsn370639-bib-0040] World Health Organisation . 2010. A Healthy Lifestyle—WHO Recommendations. https://www.who.int/europe/news‐room/fact‐sheets/item/a‐healthy‐lifestyle‐‐‐who‐recommendations.

[fsn370639-bib-0041] World Health Organisation . 2023. Saturated Fatty Acid and Trans‐Fatty Acid Intake for Adults and Children: WHO Guideline. World Health Organization. 37490572

[fsn370639-bib-0042] Zang, B. Y. , L. X. He , and L. Xue . 2022. “Intermittent Fasting: Potential Bridge of Obesity and Diabetes to Health?” Nutrients 14, no. 5: 981. 10.3390/nu14050981. 35267959 PMC8912812

[fsn370639-bib-0043] Zhang, F. , J. Ye , X. Zhu , et al. 2019. “Anti‐Obesity Effects of Dietary Calcium: The Evidence and Possible Mechanisms.” International Journal of Molecular Sciences 20, no. 12: 3072. 10.3390/ijms20123072. 31234600 PMC6627166

[fsn370639-bib-0044] Zhao, Y. , Y. Li , W. Wang , et al. 2023. “Low‐Carbohydrate Diets, Low‐Fat Diets, and Mortality in Middle‐Aged and Older People: A Prospective Cohort Study.” Journal of Internal Medicine 294, no. 2: 203–215. 10.1111/joim.13639. 37132226

[fsn370639-bib-0045] Zheng, Y. , Y. Fang , X. Xu , et al. 2023. “Dietary Saturated Fatty Acids Increased All‐Cause and Cardiovascular Disease Mortality in an Elderly Population: The National Health and Nutrition Examination Survey.” Nutrition Research (New York, N.Y.) 120: 99–114. 10.1016/j.nutres.2023.10.002. 37952265

[fsn370639-bib-0046] Zhou, H. , C. J. Urso , and V. Jadeja . 2020. “Saturated Fatty Acids in Obesity‐Associated Inflammation.” Journal of Inflammation Research 13: 1–14. 10.2147/JIR.S229691. 32021375 PMC6954080

[fsn370639-bib-0047] Zhu, H. , D. Bi , Y. Zhang , et al. 2022. “Ketogenic Diet for Human Diseases: The Underlying Mechanisms and Potential for Clinical Implementations.” Signal Transduction and Targeted Therapy 7, no. 1: 11. 10.1038/s41392-021-00831-w. 35034957 PMC8761750

